# Recommended data elements for health registries: a survey from a German funding initiative

**DOI:** 10.1186/s12911-024-02535-x

**Published:** 2024-05-27

**Authors:** Sonja Harkener, Ekkehart Jenetzky, Rüdiger Rupp, Jennifer Dell, Christoph Engel, Maximilian Ferry von Bargen, Robert Finger, Maximilian Glienke, Carsten Heinz, Patrick Jersch, David Martin, Rita Schmutzler, Martin Schönthaler, Barbara Suwelack, Jeannine Wegner, Jürgen Stausberg

**Affiliations:** 1 Faculty of Medicine, University Duisburg-Essen, IMIBE, Essen, Germany; 2 Faculty of Health/School of Medicine, Witten/Herdecke University, Witten, Germany; 3 University Medical Center of the Johannes-Gutenberg-University, Mainz, Germany; 4 Spinal Cord Injury Center, Heidelberg University Hospital, Heidelberg, Germany; 5 Department of Ophthalmology, University Hospital Bonn, Bonn, Germany; 6 Leipzig University, IMISE, Leipzig, Germany; 7 Department of Urology, Faculty of Medicine, Medical Centre - University of Freiburg, University of Freiburg, Freiburg, Germany; 8 Department of Ophthalmology, University Hospital Mannheim, Mannheim, Germany; 9 Department of Ophthalmology, St. Franziskus-Hospital Münster, Münster, Germany; 10 Department of Ophthalmology at University Essen, Essen, Germany; 11 Department of Pediatrics, Eberhard-Karls University Tübingen, Tübingen, Germany; 12 Center for Familial Breast and Ovarian Cancer, University of Cologne, Cologne, Germany; 13 Department of Medicine D, Division of General Internal Medicine, Nephrology and Rheumatology, University Hospital Muenster, Muenster, Germany

**Keywords:** Data element, Health care, Health services research, Metadata, Registry

## Abstract

**Background:**

The selection of data elements is a decisive task within the development of a health registry. Having the right metadata is crucial for answering the particular research questions. Furthermore, the set of data elements determines the registries’ readiness of interoperability and data reusability to a major extent. Six health registries shared and published their metadata within a German funding initiative. As one step in the direction of a common set of data elements, a selection of those metadata was evaluated with regard to their appropriateness for a broader usage.

**Methods:**

Each registry was asked to contribute a 10%-selection of their data elements to an evaluation sample. The survey was set up with the online survey tool „LimeSurvey Cloud”. The registries and an accompanying project participated in the survey with one vote for each project. The data elements were offered in content groups along with the question of whether the data element is appropriate for health registries on a broader scale. The question could be answered using a Likert scale with five options. Furthermore, “no answer” was allowed. The level of agreement was assessed using weighted Cohen’s kappa and Kendall’s coefficient of concordance.

**Results:**

The evaluation sample consisted of 269 data elements. With a grade of “perhaps recommendable” or higher in the mean, 169 data elements were selected. These data elements belong preferably to groups’ demography, education/occupation, medication, and nutrition. Half of the registries lost significance compared with their percentage of data elements in the evaluation sample, one remained stable. The level of concordance was adequate.

**Conclusions:**

The survey revealed a set of 169 data elements recommended for health registries. When developing a registry, this set could be valuable help in selecting the metadata appropriate to answer the registry’s research questions. However, due to the high specificity of research questions, data elements beyond this set will be needed to cover the whole range of interests of a register. A broader discussion and subsequent surveys are needed to establish a common set of data elements on an international scale.

## Background

Definition and maintenance of data elements are important tasks contributing essentially to the success of a health registry. The selection of data elements should mainly be guided by the predefined research questions on one hand. On the other hand, the data elements have to be appropriate to fulfil other requirements related to the usage of the registry, in particular to any calculations defined in its analysis plan. Consequently, definition and maintenance of data elements play a major role in recommendations about development and the operation of health registries [1–3]. “As little as possible, as much as necessary” - this rule of thumb might be a good advisor in the selection of data elements. The demands and requirements towards this selection are manifold. Reliable and valid data elements would be preferred. Available reference values for a data element would allow an external benchmarking of the registry‘s results. Data elements might be recommended by established organizations, since they might at least be in use elsewhere. Beside the mentioned aspects of necessity and suitability, the use of the data elements should also be practically feasible. Collection and recording of the information must be legally permissible, possible and justifiable. Sometimes, information might already be recorded in other data collections, such as administrative data or health records. Lastly, data elements have to be applicable for the data collection tools being used. They should be accessible for data monitoring purposes. An integration into a predefined statistical analysis plan must be supported.

Identifying necessary, suitable, feasible and implementable data elements is a challenging and time-consuming task for health registries. This process has to be balanced between individual needs of a registry and advantages of using existing recommendations for data elements, data definitions and terminological concepts. The US-American Agency for Healthcare Research and Quality (AHRQ) claims for a simplification of the data element selection process by using standards [1]. Furthermore, the use of standards can improve “the ability of the registry to compare and exchange data with other systems in the future” according to the AHRQ. Predefined collections of data elements might be helpful to facilitate this process. One example is the Set of Common Data Elements (CDE) recommended for rare disease registries [4]. This set contains 16 data elements such as date of birth and sex (cf. https://eu-rd-platform.jrc.ec.europa.eu/set-of-common-data-elements_en). The number of data elements of the CDE is surprisingly low compared to the high number of 100 and more data elements typically implemented in health registries [5]. The huge difference in magnitude of these numbers indicate challenges in the general definition of what is “common”. Attempts to map data elements between different registries failed due to the high specificity of the registries‘ particular research focuses. A comparison of 38 registries revealed that only 4 concepts (represented by different data elements), such as sex and date of birth, met the criterion of being present in 50% or more of the registries [6]. Lowering the threshold to 20%, Tcheng et al. identified 15 concepts (e.g. ethnicity).

Within the first conceptual phase of registries in health services research funded by the German Federal Ministry of Education and Research (BMBF), nearly 4,000 data elements were defined in total by 15 projects with a mean of 260 ± 195 elements (range 48 to 756) per registry [7]. Based on the identification of commonly used elements, the idea of formulating recommendations for shared data elements could only be carried out for seven data elements: sex, date of birth, number of procedures, reason for admission, pseudonym, highest school degree, and highest professional qualification. In general, supporting health registries in defining their data elements with a set of common recommendations seems to be limited.

However, data elements and their implementation might not only be of interest for each single registry. Data exchange between registries (interoperability) and access to registry data from third parties (reusability) currently receive great attention. Ideally, the definition of data elements would consider these aspects beyond the requirements of the responsible registry. Looking at the 15 FAIR Guiding Principles [8], the definition of data elements is particularly concerned with four principles:

- The definition of data elements should use a formal, accessible, shared, and broadly applicable language for knowledge representation (FAIR Guiding Principle Interoperability 1).
- The definition of metadata uses vocabularies that follow FAIR principles (Interoperability 2).
- The definition of data elements includes qualified references to other data elements (Interoperability 3).
- Data elements are well-described with a plurality of accurate and relevant attributes (Reusability 1).

With respect to these FAIR Guiding Principles, it could be helpful to offer a broad collection of data elements potentially relevant for health registries, even if those data elements do not reach the level of relevance and enforceability one would expect from a common data set. Therefore, this study aimed at a first draft of a data element collection useful for health registries. Within the German funding initiative for health services research, this draft of a consensus-based collection is proposed based on the compilation of all data elements from the six finally established registries.

## Methods

### Funding initiative

The BMBF funded the implementation of six investigator-initiated patient registries within an initiative for health services research [9]. Legal basis of all registries was an ethics vote of an approved ethics committee as well as the inclusion of patients using an informed consent. The registries were formally operated by universities, university clinics, and clinical or scientific associations. The projects started in 2019 with a funding period of three (one registry) or five years (five registries). One registry underwent an interim review after two years of funding. The registries aimed at answering very specific research questions related to their medical field of interest: spinal cord injury or disorder [10], fever episodes in children [11], treatment exit options for non-infectious non-anterior uveitis, women at risk of ovarian and breast cancer [12], patients suffering from recurrent calculi of the urinary tract [13, 14], and living donors of kidney transplants [15]. In 2022, the registries recruited between 224 and 17,468 participants with a median of 991 patients. One registry has not started the recruitment so far. An accompanying project supported the six registries in establishing methodical, technical and structural standards. The presented survey was organized by this project.

For this work, a compilation of the registries’ data elements updated in the beginning of 2023 was used. This set of data elements is maintained by the accompanying project in a metadata repository implemented with Microsoft Access. The compilation is available for download in German from the Working Group Registries of the non-profit association German Network Health Service Research (cf. https://www.dnvf.de/groups/ag-register.html). The compilation includes a total of 2,463 data elements with a range between 121 and 865 data elements per registry (median 468.5). From the 2,463 data elements, 999 are defined through a categorical value domain (40.6%). The others are tagged with a data type derived from HL7 FHIR Release 5 (cf. https://www.hl7.org/fhir/datatypes.html). Value domains representing large coding systems as the International Statistical Classification of Diseases and Related Health Problems (ICD) were defined as external references.

### Definitions

A consented terminology is in general missing in the field of data management [16], which includes also the terms “data element” and “value domain”. We refer to the view provided with the metamodel of ISO/IEC 11179 Information technology — Metadata registries (MDR) in its third edition [17]. ISO/IEC 11179 defines a data element as a unit of data that is considered in context to be indivisible, a value domain as a set of permissible values. The data element is constructed through the combination of a data element concept as “sex of a patient” with a value domain with permissible values as male or female. The data element is therefore a fixed binding of a data element concept with a value domain. Counterpart of the data element on a conceptual level is the conceptual domain. This allows the mapping of similar data elements which differ, for example, only in the coding of permissible values. To achieve a higher level of aggregation, we added a conceptual domain group to the ISO/IEC 11179 information model. This allowed us to organize the data elements of our compilation in a highly structured manner. Then, we used the term “metadata” for the set of all data elements of a data collection.

**Fig. 1:**
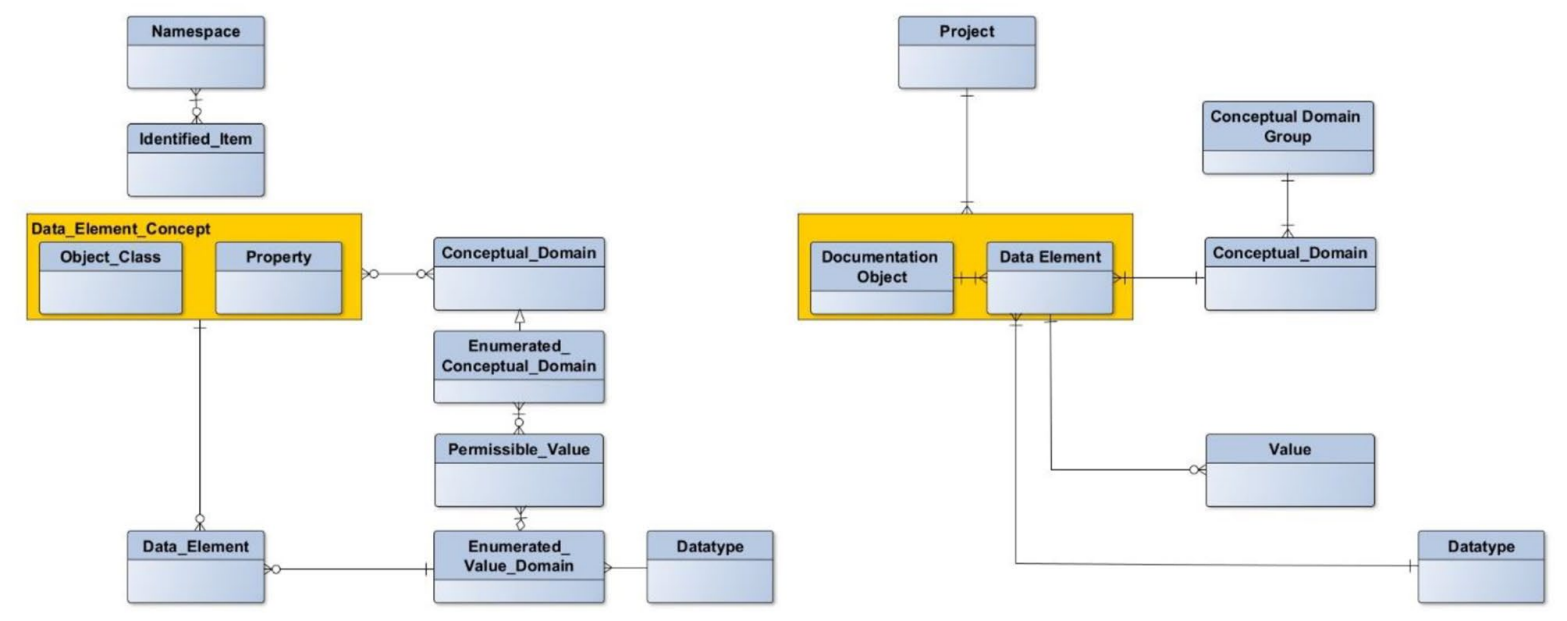
Mapping of the catalog of data elements (right) to the metamodel of ISO/IEC 11179 (left) adopted from [7]

In the initial phase of registry development, the accompanying project recommended a structure for a catalog of data elements that registries could use to define their metadata [18]. This approach was based on the work of Leiner and Haux who introduced a documentation protocol with a documentation scheme [19]. Some, but not all registries used this recommendation. Others applied individual or tool-based structures for the definition of their metadata. The understanding of core terms such as data element differed between the registries as well [20]. Therefore, the registries’ metadata were first transferred into the recommended structure of a catalog of data elements and then embedded into the metamodel of ISO/IEC 11179. Fig. 1 shows the final structure of our compilation. Each data element is mandatorily assigned to exactly one registry. Therefore, some entries appeared multiple times, e.g. date of birth. The class Conceptual_Domain_Group was added as additional clustering level. An Enumerated_Conceptual_Domain class is indicated through the datatype “coding”.

### Survey

Due to the high specificity of the registries’ metadata, we decided to include only a 10%-sample of data elements from the compilation into our survey. It was up to the registries to select data elements with high relevance for other registries. We ended up with 269 data elements (10.9% from overall 2,463 data elements). We denote this compilation of 269 data elements “evaluation sample” in the following.

The registry with the topmost number of total data elements submitted only 3.5% of their total data elements to this survey, the others submitted between 11.6% and 22.1%. The proportion of data elements with a categorical value domain was 43.9% (118 from 269 data elements), slightly higher than in the whole compilation with 40.6%. The sample covered 31 from 39 conceptual domain groups (79.5%) and 149 from 408 conceptual domains (36.5%).

The survey was implemented with the online survey tool „LimeSurvey Cloud” offered by LimeSurvey GmbH (cf. https://www.limesurvey.org/). The survey was structured into 31 pages representing the conceptual domain groups of the 269 data elements in the sample (cf. Table 1 for the list of conceptual domain groups). The range of data elements per page was one to 29 with a median of five data elements. For each data element, the following information was displayed:

**Table 1:** Distribution of data elements among the conceptual domain groups

Conceptual domain group	Total data elements		Evaluation sample		Recommended set	
	*N*	%	*N*	%	*N*	%
Alcohol/Dependence causing substance	7	0.3%	6	2.2%	1	0.6%
Application program - App	18	0.7%	2	0.7%	1	0.6%
Breast cancer	3	0.1%	0	0.0%	0	0.0%
Classification (e.g. ICD)	16	0.6%	7	2.6%	5	3.0%
Clinical trial	3	0.1%	0	0.0%	0	0.0%
Comment	9	0.4%	1	0.4%	0	0.0%
Complication/Adverse reaction	34	1.4%	2	0.7%	2	1.2%
Consultation	6	0.2%	0	0.0%	0	0.0%
Contact with institution	31	1.3%	10	3.7%	10	5.9%
Corona pandemic	10	0.4%	3	1.1%	0	0.0%
Death	8	0.3%	4	1.5%	3	1.8%
Demography	64	2.6%	26	9.7%	20	11.8%
Diagnosis/Disease/Symptom	446	18.1%	26	9.7%	8	4.7%
Disease risk	4	0.2%	0	0.0%	0	0.0%
Education/Occupation	17	0.7%	14	5.2%	14	8.3%
Examination	79	3.2%	3	1.1%	2	1.2%
Family	11	0.4%	5	1.9%	4	2.4%
Genetics	47	1.9%	22	8.2%	0	0.0%
Health insurance	5	0.2%	2	0.7%	2	1.2%
Health status	5	0.2%	1	0.4%	1	0.6%
Institution	46	1.9%	12	4.5%	7	4.1%
Intensified screening program (ISP)	9	0.4%	0	0.0%	0	0.0%
Living conditions	2	0.1%	2	0.7%	2	1.2%
Localization	28	1.1%	8	3.0%	3	1.8%
Management of the data collection	96	3.9%	14	5.2%	2	1.2%
Medication	716	29.1%	24	8.9%	17	10.1%
Nursing	4	0.2%	0	0.0%	0	0.0%
Nutrition	29	1.2%	29	10.8%	29	17.2%
Operation	31	1.3%	2	0.7%	0	0.0%
Organ donation	28	1.1%	0	0.0%	0	0.0%
Participation in the registry	38	1.5%	10	3.7%	8	4.7%
Pregnancy	7	0.3%	2	0.7%	2	1.2%
Questionnaire/Score/Scale	190	7.7%	10	3.7%	10	5.9%
Reason	1	0.0%	0	0.0%	0	0.0%
Self-assessment	2	0.1%	1	0.4%	1	0.6%
Smoking	2	0.1%	2	0.7%	2	1.2%
Technical equipment	2	0.1%	2	0.7%	0	0.0%
Therapy/Procedure	215	8.7%	3	1.1%	2	1.2%
Value/Finding/Result	194	7.9%	14	5.2%	11	6.5%
**All**	**2,463**	**100.0%**	**269**	**100.0%**	**169**	**100.0%**

- conceptual domain the data element belongs to,
- denomination of the data element,
- free text description of the data element if available,
- data type,
- list of values in case of categorical value domains.

The submitting registry was not cited. There was only one question to be answered by the raters: “Could this data element be recommended for registries other than the registries in the funding scheme?” A Likert scale with five options was used for grading the assessment: no way, rather not, maybe, rather yes, for sure. „No response” was additionally used as a default option. Furthermore, a free text box for comments was available. Intermediate results could be saved, the survey could be interrupted, and the editing could be split up to several persons. Instructions were available separately from the LimeSurvey Cloud as a PDF-file. After completion of the survey, the assessments could be either saved or printed. A modification was not possible after this stage. A prerequisite for the participation was active consent to the privacy statement of the survey. The survey started on June 26, 2023 and ended on September 5, 2023.

All six registries were invited for the survey, an additional vote was possible by the accompanying project. The access to the survey was based on the project’s identity and could be shared between different individuals. The projects themselves decided on the persons performing the assessments. For analysis, no information beyond the projects’ identities was available. In total, seven votes could be included in the analysis independently from the number of persons involved in the project’s individual assessments. For five registries, clinicians with a university professorship were in charge of the project responsibility, in one registry an engineer with a university professorship, for the accompanying project a medical specialist for medical informatics.

### Statistics

Every project had exactly one vote for a data element. Votes for data elements of the own registry were excluded in the analysis resulting in a maximum of six votes for each data element and a total number of 1,614 votes (269 data elements multiplied with seven projects minus 269 data elements to avoid self-evaluation). The five options were coded with 1 (no way) to 5 (for sure). A recommendation of a data element was concluded in case of an arithmetic mean rating > = 3 (i.e. a sum of 18 and higher), including answers with the option “no response” in the denominator. The remaining excerpt from the evaluation sample is denoted as “recommended set” in the following.

The level of concordance was measured using weighted Cohen’s kappa and Kendall’s coefficient of concordance (Kendall’s W). On one hand, we calculated mean and standard deviation of the weighted kappa for all combinations of two raters (seven raters, 21 combinations of two raters). On the other hand, we calculated mean and standard deviation of Kendall’s W for the six registry samples of data elements. Both calculations were repeated with and without votes of “no response”, in the given case coded with “0”. We interpreted the results according to the grading recommended by Landis and Koch [21]: poor - <0.00, slight − 0.00-0.20, fair − 0.21–0.40, moderate − 0.41–0.60, substantial − 0.61–0.80, almost perfect 0.81-1.00.

The survey results were exported from the LimeSurvey Cloud and managed with Microsoft Access 2013. Descriptive figures were derived using Microsoft Access 2013 and Microsoft Excel 2013. IBM SPSS Statistics Version 28 was used to calculate Cohen’s kappa and Kendall’s W.

## Results

### Agreement of raters

In total, 1,614 votes were recorded from seven projects. In 296 votes, the raters choose “no response” (18.3%). Mean weighted kappa was 0.19 ± 0.141 considering “no response” and 0.12 ± 0.091 without considering “no response”. The mean of Kendall’s W was 0.29 ± 0.097 considering “no response” and 0.31 ± 0.128 without considering “no response”. According to Landis and Koch, the agreement could be assessed as being fair.

### Composition of the recommended set

**Fig. 2:**
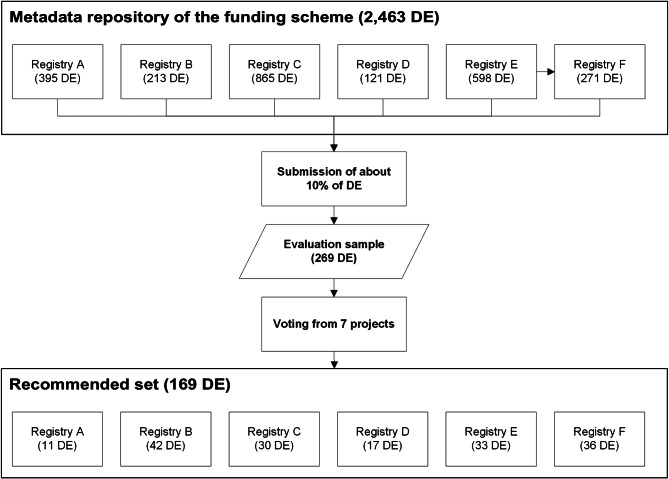
Number of data elements (DE) in the survey

Out of the evaluation sample of 269 data elements (Fig. 2), 169 data elements were selected in the survey with a mean rating of 3 and higher (62.8%). The highest possible mean rating of 5 was assigned to 9 data elements (5.3% from 169 data elements), 61 data elements had a mean of 4 to less than 5 (36.1%), and 99 data elements had a mean rating of 3 to less than 4 (58.6%). The proportion of data elements with a categorical value domain increased from 40.6% in the total sample to 50.9% in the recommended set (89 from 169 data elements). The qualified data elements belonged to 95 conceptual domains, 23.3% of all 408 conceptual domains used in the metadata repository.

The nine data elements with a rating of 5 were spread over only four conceptual domain groups (Table 2). Three of these nine data elements belonged to the conceptual domain group “Participation in the registry” (33.3%), another three to the conceptual domain group “Value/Finding/Result” (33.3%). Only one of the nine data elements had a categorical value domain (data element “Pregnancy”).

**Table 2:** Data elements with an optimal rating

Conceptual domain group	Denomination^#^	Value domain
Demography	Year of birth	time/date/dateTime
	Year of birth	time/date/dateTime
Participation in the registry	Date of consent for study participation	time/date/dateTime
	End of study participation	time/date/dateTime
	Patient consent is available	boolean
Pregnancy	Pregnancy	present|not present|unknown
Value/Finding/Result	Body height in cm	numerical
	Height	numerical
	Weight in kg	numerical

^*#*^ *Data elements with an identical or comparable denomination belong to different registries.*

None of the data elements from the conceptual domain groups “Comment”, “Corona pandemic”, “Genetics”, “Operation” and “Technical equipment” were rated as commonly relevant to registries (Table 1). Furthermore, the representation of the conceptual domain groups changed significantly between the total of all data elements, the evaluation sample and the selection of recommended data elements. In three conceptual domain groups related to social habits, all data elements were kept during the selection process, living conditions (2 data elements), nutrition (29), and smoking (2). Nutrition-related data elements gained relevance starting from position 15 in the metadata repository (29 data elements, 1.2%) to the conceptual domain group with the most data elements in the recommended set (29, 17.2%). The greatest loss of relevance occurred in the conceptual domain groups “Diagnosis/Disease/Symptom” (18.1% of the data elements in the metadata repository, 4.7% of the data elements in the recommendation), “Medication” (29.1%, 10.1%), and “Therapy/Procedure” (8.7%, 1.2%). These three conceptual domain groups covered mostly data elements highly specific for the individual population in the focus of each registry. Some conceptual domains were overrepresented in the evaluation sample, without retaining this focus in the survey as “Alcohol/Dependence causing substance”, “Localization”, and “Management of the data collection”. The proportion of data elements from the conceptual domain groups “Complication/Adverse reaction”, “Medication”, “Questionnaire/Score/Scale”, and “Value/Finding/Result” increased from 18.6% in the evaluation sample to 23.7% in the recommended set.

### Distribution of data elements per registry

Looking at the results from the perspective of the registries (Table 3), between 23 and 84 data elements were selected for evaluation, whereby the share of the data elements chosen in the registry ranges between 3.5% and 22.1% (10% should be targeted). Within the evaluation sample, registries are represented with shares between 8.6% and 31.2%. In the recommended set, the distribution is in a similar range (6.5–24.9%), but differs among the registries. Thus, the shares are lower in the recommended set than in the evaluation sample for two registries (17.1–6.5% and 31.2–19.5%, respectively), and higher for four registries than in the sample for evaluation (24.9–17.5%, 17.8–11.2%, and 21.3–14.5%).

**Table 3:** Distribution of data elements among the registries

Registry	Total data elements			Evaluation sample			Recommended set		
	*N*	%	% row^#^	*N*	%	% row^#^	*N*	%	% row^#^
A	395	16.0%	100.0%	46	17.1%	11.6%	11	6,5%	2,8%
B	213	8.6%	100.0%	47	17.5%	22.1%	42	24,9%	19,7%
C	865	35.1%	100.0%	30	11.2%	3.5%	30	17,8%	3,5%
D	121	4.9%	100.0%	23	8.6%	19.0%	17	10,1%	14,0%
E	598	24.3%	100.0%	84	31.2%	14.0%	33	19,5%	5,5%
F	271	11.0%	100.0%	39	14.5%	14.4%	36	21,3%	13,3%
**All**	**2,463**	**100.0%**	**100.0%**	**269**	**100.0%**	**10.9%**	**169**	**100.0%**	**6.9%**

^*#*^ *% row denotes the percentage in relation to the total number of data elements.*

From the evaluation sample of four registries, more than 70% of the data elements were considered in the recommendation set. It is particularly noticeable that all data elements of the registry (registry C in Table 3) that had contributed the smallest number of data elements from its own metadata for the selection (3.5%) were chosen for the recommended set. If the data elements with the highest recommendations are considered, this registry also has the highest share (7 out of 9) of data elements with a mean of 5 (77.8%).

## Discussion and conclusions

To the best of our knowledge, we present the first set of recommended data elements for patient registries in health services research, at least for Germany. The set includes 169 data elements from six registries interested in different medical conditions in health services research. The data elements originated from real world data studies and represent material used in daily management of case reporting. The data elements were taken at face value without any curation. The set covers a broad range of topics relevant for patient registries, starting with the management of the data collection and demographics up to educational and job-related issues. Presumably, the data elements covered by the conceptual domain group “Genetics” were too specific to be recommended from the broader perspective that lead the selection process. The raters preferred data elements with a categorical value domain. Having at least 50% of categorical data elements could be a valuable benchmark for high quality metadata.

Our selection should neither be regarded as a minimal data set nor as a complete collection of all possible elements. According to our experience, patient registries rely on specific data elements depending on the conditions and research questions they are interested in. Our compilation should be rather taken as a supportive measure within the systematic process of developing a registry [1]. A selection of data elements for a patient registry should be triggered by predefined research questions on one hand and the necessities of the planned statistical analysis on the other hand. However, our consensus-based compilation of real-world implemented data elements could be very helpful for the fine tuning of a registries’ metadata. As pointed out by the AHRQ [1], most registries will have to develop some data elements and data definitions themselves, because our compilation as well as other lists of data elements do not fully meet their needs. But registries should not reinvent the wheel if their needs are already covered.

There is a huge overlap between the conceptual domain groups remaining in our recommendation and the 15 classes of common clinical concepts proposed by Tcheng et al. [6]. However, the designations deviate. Data elements related to demographical information are broadly considered in both sets. The same holds true for data elements representing information about vital signs, laboratory results, and medications covered by the conceptual domain groups “Medication” and “Value/Finding/Result”. Smoking and vital status are present in both sets. Only one data element regarding substance use did qualify in our survey, a domain recommended by Tcheng et al. Furthermore, data elements related to other procedures than the ones mentioned before are underrepresented in our recommended set.

The data elements for our recommendation were selected in a two-step process. In the first step, it was up to the registries to deliver a 10% sample from their metadata which they rated relevant for other registries. In the second step, the registries judged the data elements of this evaluation sample concerning their applicability for a broader use. Nearly two thirds of the data elements of the evaluation sample qualified for the recommendation. This high percentage confirms a careful selection process by the registries and the relevance of the submitted samples. As a conclusion, we do not expect that important data elements were not considered on a broader scale. The selection of data elements for the survey shows a good assessment of the registries with regard to their usability in other registries. However, with regard to the specific data elements on the topic of genetics there was a reluctance from one registry to make a recommendation.

Interestingly, changes in the shares of the registries’ metadata suggest an opposing effect. The data elements of the three registries represented in the metadata repository with a number of data elements above the median of 333 data elements lost significance in the recommended set. The other three registries with a number of data elements below the median gained significance. This might be an indicator for a restrictive inclusion of data elements into a registry’s metadata based on research questions and necessities of the analyses, as mentioned in the introduction. Additionally, a particularly careful consideration of categorical data elements might be advisable. Data elements with a value domain of this type were preferred by the raters in our study.

The concordance of ratings was calculated as being fair. To some extent, the low degree of agreement could be explained through different interpretations of the concept of a data element leading to quite different types of denominations [20]. Some registries used abbreviations as denomination of a data element, other registries used labels, terms or questions. We agree with Tcheng et al. in the importance of a rich set of attributes that is needed to fully describe a data element and the context it belongs to. In our survey, we displayed a description for a data element provided by the registries and the conceptual domain group, which was allocated to the data element by the accompanying project. It is future work to consent on the appropriate set of attributes needed to fully describe a data element. Then, the FAIR Guiding Principle R1 might be satisfied, to richly describe data elements with a plurality of accurate and relevant attributes [8].

The missing control over the individuals performing the assessments on behalf of the projects might be a limitation of the survey. It was the principal investigator’s responsibility to decide about the project’s approach. For some projects, an individual might have done the assessment, for other projects, the assessment might reflect an internal consent based approach. However, we think that the assessment was mainly performed by persons involved in the registries’ metadata definition and management. All raters were familiar with the principles and ideas behind the survey. The structure used for the presentation of data elements and the mapping of the projects’ metadata to the metadata model of ISO/IEC 11179 were introduced in the funding initiative before and independently of the survey. We do not know whether the assessment was biased through the alphabetical ordering of conceptual domain groups, conceptual domains, and data elements in the survey. On the one hand, this ordering might disclose relations between data elements that could be helpful to derive a valid assessment. On the other hand, the level of assessment might be influenced through fatigue symptoms or an experience gain. It might be worthwhile to consider a random sequence in subsequent work.

The German funding initiative offered a unique opportunity to analyze, to discuss and to evaluate data elements used in health registries. The recommended set of data elements is published - as the whole compilation of the registries metadata - for download in German as PDF-file at https://www.dnvf.de/files/theme_files/pdf/PDF-AG_Register/metadaten_ausschnitt_20230914.pdf. Our recommendation could be a starting point for a broader initiative aiming at the establishment of a consented set of data elements for health registries on an international level. Particularly, the recommended set would benefit from the additon of further contributions from other medical fields.

## Data Availability

No datasets were generated or analysed during the current study.

## References

[1] Glicklich RE, Leavy MB, Dreyer NA, editors. Registries for evaluating patient outcomes: a user’s guide. 4th ed. AHRQ Publication No. 19(20)-EHC020. Rockville, MD: Agency for Healthcare Research and Quality; September 2020. 24945055

[2] Stausberg J, Maier B, Bestehorn K, Gothe H, Groene O, Jacke C, Jänicke M, Kostuj T, Mathes T, Niemeyer A, Olbrich K, Schmitt J, Neugebauer E (2020). Memorandum Registry for Health Services Research: Update 2019. Das Gesundheitswesen.

[3] Zaletel M, Kralj M (2015). Methodological guidelines and recommendations for efficient and rational governance of patient registries.

[4] Taruscio D, Mollo E, Gainotti S, Posada de la Paz M, Bianchi F, Vittozzi L (2014). The EPIRARE proposal of a set of indicators and common data elements for the European platform for rare disease registration. Archives PublicHealth.

[5] Stausberg J, Altmann U, Antony G, Drepper J, Sax U, Schütt A (2010). Registers for networked medical research in Germany. Situation and prospects. Appl Clin Inf.

[6] Tcheng JE, Drozda JP, Gabriel D, Heath A, Wilgus RW, Williams M, Windle TA, Windle JR. Achieving Data Liquidity: Lessons Learned from Analysis of 38 Clinical Registries (The Duke-Pew Data Interoperability Project). AMIA Annu Symp Proc. 2020; 2019: 864–873. PMC7153125 32308883

[7] Stausberg J, Harkener S (2021). Metadata of registries: results from an initiative in health services research. Stud Health Technol Inf.

[8] Wilkinson MD, Dumontier M, Aalbersberg IJ, Appleton G, Axton M, Baak A (2016). The FAIR Guiding principles for scientific data management and stewardship. Sci Data.

[9] Stausberg J, Harkener S, Semler S (2021). Recent trends in patient registries for health services research. Methods Inf Med.

[10] Rupp R, Jersch P, Schuld C, Schweidler J, Benning, Knaup-Gregori, Aach, Badke, Hildesheim, Maier D, Weidner, Saur (2021). Germany-wide, web-based ParaReg Registry for lifelong monitoring of people with spinal cord Injury: Data Model, Ethico-legal prerequisites and technical implementation. Gesundheitswesen.

[11] Martin D, Wachtmeister J, Ludwigs K, Jenetzky E (2020). The FeverApp registry - ecological momentary assessment (EMA) of fever management in families regarding conformity to up-to-date recommendations. BMC Med Inf Decis Mak.

[12] Engel C, Wieland K, Zachariae S, Bucksch K, Enders U, Schoenwiese U, Yahiaoui-Doktor M, Keupp K, Waha A, Hahnen E, Remy R, Ernst C, Loeffler M, Schmutzler RK (2021). HerediCaRe: documentation and IT solution of a Specialized Registry for Hereditary breast and ovarian Cancer. Gesundheitswesen.

[13] Schoenthaler M, Fichtner UA, Boeker M, Zoeller D, Binder H, Prokosch HU, Praus F, Walther T, Glienke M, Horki P, Gratzke C, Farin-Glattacker E (2022). A nationwide registry for recurrent urolithiasis in the upper urinary tract - the RECUR study protocol. BMC Health Serv Res.

[14] Walther T, Farin E, Boeker M, Prokosch HU, Binder H, Praus F, Ploner N, Fichtner UA, Horki P, Haeuslschmid R, Seuchter S, Gratzke C, Schoenthaler M (2021). RECUR - establishment of an Automated Digital Registry for Patients with recurrent stones in the Upper urinary tract. Gesundheitswesen.

[15] Brix TJ, Greulich L, Janssen A, Riepenhausen S, Neuhaus P, Oehm J, Wegner J, Suwelack B, Storck M, Varghese J (2022). Linking EMR Data to REDCap: implementation in the SOLKID Register. Stud Health Technol Inf.

[16] Tahar K, Martin T, Mou Y, Verbuecheln R, Graessner H, Krefting D (2023). Rare diseases in Hospital Information Systems-An interoperable methodology for distributed data quality assessments. Methods Inf Med.

[17] ISO/IEC (2013). ISO/IEC 11179-3. Information technology — metadata registries (MDR) — part 3: Registry metamodel and basic attributes. Third edition 2013-02-15.

[18] Stausberg J, Harkener S (2019). Bridging documentation and metadata standards: experiences from a funding initiative for registries. Stud Health Technol Inf.

[19] Leiner F, Haux R (1996). Systematic planning of clinical documentation. Methods Inf Med.

[20] Stausberg J, Harkener S, Burgmer M, Engel C, Finger R, Heinz C, Jenetzky E, Martin D, Rupp R, Schoenthaler M, Schuld C, Suwelack B, Wegner J (2022). Metadata Definition in registries: what is a data element?. Stud Health Technol Inf.

[21] Landis JR, Koch GG (1979). The measurement of observer agreement for categorical data. Biometrics.

